# Standardized immunological assays for assessing COVID-19 vaccines by the CEPI-Centralized Laboratory Network

**DOI:** 10.1038/s41541-024-00921-0

**Published:** 2024-07-16

**Authors:** Ali Azizi, Deborah Ogbeni, Gathoni Kamuyu, Lauren M. Schwartz, Carolyn Clark, Peter Spencer, Valentina Bernasconi

**Affiliations:** 1 CEPI, 1901 Pennsylvania Ave, NW #1003, Washington, DC USA; 2 CEPI, Gibbs Building, 215 Euston Rd, Bloomsbury, London, UK; 3 Gorman Consulting, 707 Driftwood Lane, Edmonds, WA USA; 4 CEPI, Skøyen Atrium, Askekroken 11, Oslo, Norway

**Keywords:** Vaccines, Diseases

## Abstract

The CEPI Centralized Laboratory Network implemented key steps in the transfer and monitoring of the developed immunological SARS-CoV-2 assays to ensure standardization across all the facilities of the network. This comprehensive evaluation reinforces the reliability of the generated data and establishes a solid foundation for a standardized approach, enabling precise inter-laboratory comparisons and contributing to the overall integrity of our network’s clinical results. Herein, we will provide a brief elaboration on the specific measures and procedures implemented to standardize the transfer of assays across our network.

## Introduction

During the SARS-CoV-2 pandemic, the absence of standardization in assay design, test platforms, viral targets, instrumentation, staff expertise, and quality systems posed a significant challenge. Therefore, in March 2020, the Coalition for Epidemic Preparedness Innovations (CEPI) initiated the Centralized Laboratory Network (CLN), a network of facilities dedicated to supporting vaccine clinical trials with immunological testing services^1–3^. To date, CEPI-CLN has contributed to the development of several approved SARS-CoV-2 vaccines (e.g., NDV-HXP-S COVID-19 vaccine produced by Thailand’s Government Pharmaceutical Organization (GPO), by testing over 100,000 sample runs from clinical trials of various vaccine developers. In this report, we outline the steps taken in the transfer and monitoring of the developed assays to achieve standardization within our network.

## Results

### Standardized processes for immunological assay development and monitoring at CEPI-CLN

Our efforts at CEPI-CLN have been structured into four key processes to ensure the standardization of immunological assays: (i) Robust assay development, validation, and tech-transfer procedures, (ii) Utilization of well-characterized assay controls and reference standards, (iii) Regular monitoring of assay performance facilitated by a centralized database, and (iv) Participation in external quality assessment programs (EQA). Herein, we briefly discuss each major process that was followed at CEPI-CLN.

(i) Assay development, validation, and tech-transfer procedures. The CEPI-CLN employed three ELISAs (Spike (S), Receptor Binding Domain (RBD), and Nucleocapsid (N)), a Microneutralization (MNA), a Pseudotyped virus-based neutralization (PNA), and an IFN-γ/IL-5 T-cell ELISpot assay to assess the immunogenicity of candidate SARS-CoV-2 vaccines globally. Initially, assay development and validations were conducted by Nexelis (now part of Q2 Solutions) and UK Health Security Agency (UKHSA) in accordance with various international guidelines including the International Council of Harmonization (ICH), European Medicines Agency’s (EMA)^4^, Food and Drug Administration (FDA) guidelines^5^, World Health Organization (WHO) technical report series^6^, and the United States Pharmacopeia (USP)^7,8^ to demonstrate suitability and stability of key reagents, evaluate assay precision, linearity, accuracy, and robustness, and establishing upper and lower limits of quantitation. In addition, we conducted a technical advisory meeting with the EMA to ensure the acceptability of our approaches in the development, validation, and transfer of assays. The performance of all assays showed suitable consistency, precision, and accuracy^1,9^. The assays were initially designed based on the Victoria variant. However, we also demonstrated strong cross-reactive immune responses when tested with samples collected following Alpha, Beta, Gamma, and Delta variants infections in the ELISA, PNA, and ELISpot assays. For the MNA, in addition to the Wuhan variant, the assay was adapted to facilitate testing for the Alpha, Beta, Gamma, Delta, Omicron BA1, BA5, and XBB.1.5 variants.

To guarantee the proper transfer of SARS-CoV-2 assays, six facilities within the CEPI-CLN underwent a comprehensive gap analysis, inter-lab studies, and re-validation for each assay. Gap analyses were performed by the reference facility prior to the initiation of assay transfer based on information provided by the receiving facility. Each exercise examined (i) equivalence of quality management systems; (ii) staff competency and know-how, which was further examined by “mock runs” to demonstrate proficiency prior to formal validation; (iii) availability of GxP status of analytical software and data analysis protocols. An example of a typical gap was the lack of proper-compliant software for analyzing the results in the binding assay. The identified gaps concerning infrastructure, logistics, quality, and scientific parameters were meticulously documented by the reference facility and subsequently shared with the receiving facility and CEPI-CLN team. The CEPI played a pivotal role in addressing some of these challenges. For instance, a budget was allocated to the receiving facility to facilitate the purchase of essential instruments, software, and recruitment of new personnel^1,9^. For the inter-lab study, Q2 Solutions-Nexelis and UKHSA provided a minimum of 40 different human samples for each assay to ensure that the receiving facility could accurately replicate the reference facility’s processes, maintaining the reliability and quality of test results. After the completion of the inter-lab study, every assay underwent a re-validation process within the receiving facility. This re-validation was undertaken to ensure that each assay met the pre-defined acceptance criteria set forth by the reference facility^1,9^. The objective was to confirm the consistent and accurate performance of the assays in the new environment, validating their reliability and adherence to quality standards in the receiving facility. This process will be extended to the more recent labs joining the CEPI-CLN, bringing the total number of labs to 17 facilities worldwide, and making it the largest network globally (https://cepi.net/charles-river-laboratories-joins-cepis-global-vaccine-assessment-network).

(ii) During and after the assay transfer, the reference facility provided critical reagents to the receiving facility. For example, the reference facility provided the coating antigen for ELISA, and the receiving facility coated the plates according to the SOP, which had been previously reviewed by the reference facility. An initial bridging experiment of the reagent is carried out at the reference facility, followed by a subsequent bridging experiment at the receiving lab whenever a new lot of a critical reagent, such as coating antigen, conjugate, blocker, or other critical materials is generated. This ensures its comparability to the reference reagent. Utilization of well-characterized assay controls and reference standards.

To achieve standardization, CEPI has partnered with the UK Medicines and Healthcare products Regulatory Agency (MHRA, formerly NIBSC) to generate well-characterized traceable controls and reference materials for the CEPI-CLN. MHRA has generated two positive controls, a low (product# 21/362) and a high (product# 21/364) control composed of one and two convalescent plasma samples, respectively, and a reference standard (product# 21/360) composed of a pool of 14 convalescent plasma samples. These products have been used as controls and reference standards for the developed antibody binding assays and neutralization assays across the network.

**Fig. 1:**
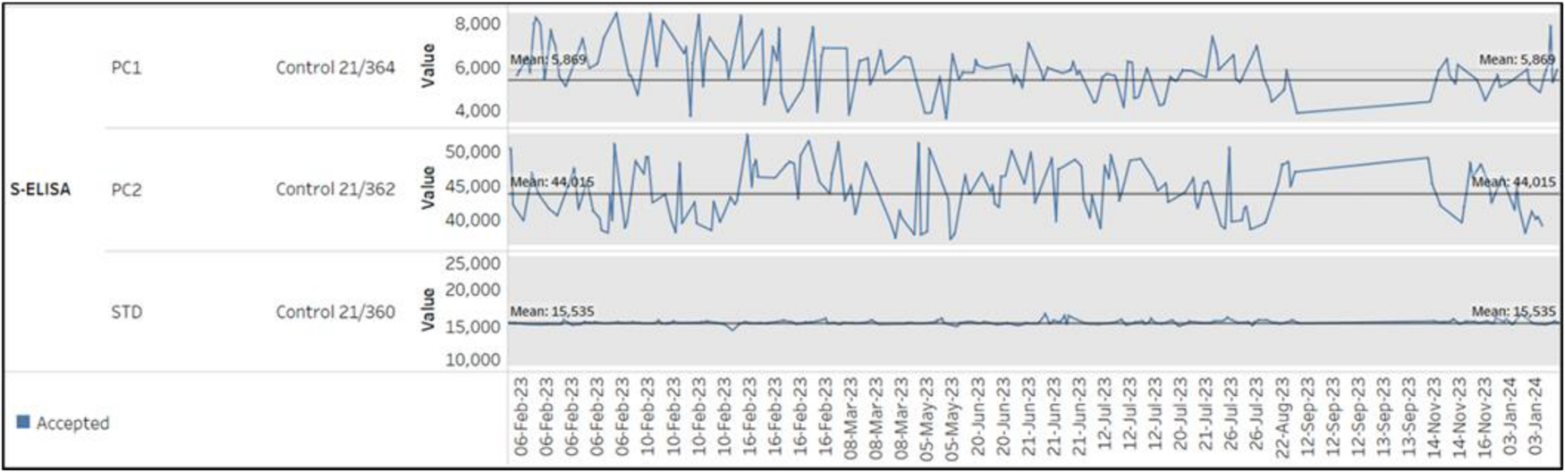
Trends in S-ELISA reference standard and controls. The trends in the reference standard (21/360) and high and low controls (21/364 and 21/362) for S-ELISA in one of the CLN facilities between February 2023 and January 2024 are presented.

Furthermore, we took a proactive approach to explore the value assignment and range determination of the SARS-CoV-2 reference standard and controls for each assay. In our pursuit of assigning a comparable value range across facilities within our network, rigorous assessments of the reference standard (21/360) and controls (21/362 and 21/364) were conducted at four facilities (Translational Health Science and Technology Institute: THSTI in India, Vismederi in Italy, Cerba Research (formerly Viroclinics) in The Netherlands, and International Centre for Diarrheal Disease Research: Icddrb in Bangladesh) within the network. The generated reference standard 21/360 and low and high controls 21/362 and 21/364 for ELISA (S, RBD, and N), MNA, and PNA assays, underwent testing by at least two analysts over multiple days in each facility. To ensure robust data, each facility generated a minimum acceptance of 14 values for the reference standard and each control for every assay. The acceptance ranges for controls and the reference standard were meticulously defined for each assay category. These specified acceptance ranges have been adopted and adhered to by all facilities within the network. Regular monitoring of assay performance facilitated by a centralized database.

(iii) A centralized database to facilitate assay data collection and quality assessment, storage and data submission was developed by an external partner, Gorman consulting. Assay output is uploaded by the facility using controlled report forms, that incorporates the pre-defined assay acceptance criteria. The report forms are created, tested & released across the network by an external partner, Gorman Consulting. The facilities test pre-clinical and clinical samples as per each vaccine developer request. The results are entered into the report forms and include the sample ID information as transferred during accessioning, date, test name, raw quantitation values, and calculated titers as interpreted by the pre-established GxP software analysis pipelines. Each facility employs quality control (QC) processes to ensure that the test results are valid, and that data are only reported from plates where standards and controls meet the specified acceptance criteria as per their SOPs. These SOPs define the criteria for when an individual plate failure or a change in assay trending over time triggers a deviation investigation. In instances where such deviations occur, facilities follow their corrective and preventive action (CAPA) system to address the issue. Investigation of one such incident of persistent N-ELISA assay failure by a member facility identified the root cause was use of an incorrect control with a different acceptance range. Following the investigation, a comprehensive preventive action plan was developed and put into effect to mitigate the risk of similar incidents occurring in the future. Further oversight is provided by the Quality Assurance (QA) function of each facility, which assures that testing and reporting is conducted in accordance with the standardized SOPs and applicable quality standards. The report forms also include formulas to convert results into international units and calculate if control values fall within expected ranges of each lot. The performance of the reference standard and controls on each plate in each facility is regularly monitored not only by the testing facility but also through a constructed internal database by CEPI-CLN staff, ensuring consistent results between facilities. As an example, the S-ELISA trends (reference standard and controls) between Feb 2023 and Jan 2024 from one of the CLN facilities are presented in Fig. 1. Instances outside the acceptance range indicate failed runs necessitating repetition. Trends of these controls are assessed by each facility, and across all facilities by CEPI, to ensure consistency in results over-time and across all studies. These approaches ensure consistency and reliability across diverse facilities, fostering a cohesive analytical framework. By employing the established acceptance ranges, we are not only monitor each assay but also fortify the comparability of clinical samples results obtained from different facilities.

**Fig. 2:**
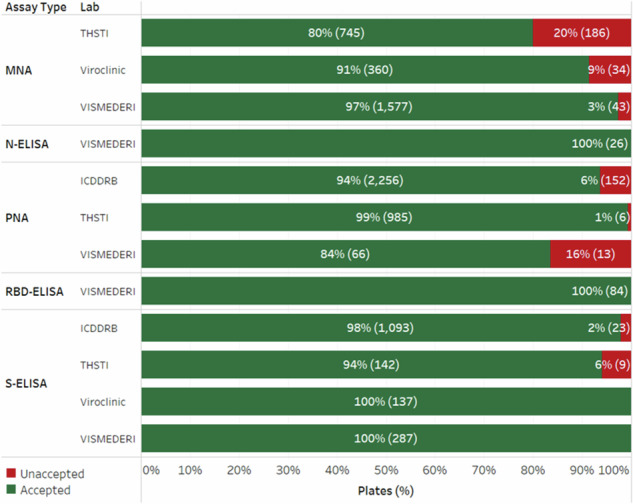
Percentages of passed plates for different assays in various facilities. The percentage of passed plates for each assay is shown in a few active testing facilities during the SARS-CoV-2 pandemic. Actual numbers of plates tested are shown in parentheses.

After the successful transfer of assays and during the actual testing of SARS-CoV-2 vaccine trial samples, we monitored the number of plates that meet the defined test and sample criteria in each testing facility. As shown in Fig. 2, the testing facilities within our network have demonstrated a noteworthy percentage of passed plates for each assay (80–100%). This indicates the robustness and effectiveness of the transferred assays in accurately and reliably processing SARS-CoV-2 samples across the network.

As a global network, International Council for Practice^10^ is Harmonizing unifying guideline for analysis of samples from clinical trials. Under this guideline, facilities are obligated to ensure that all clinical trial data, including those in electronic format, are recorded, handled and stored in a way that protects their integrity. Facilities retain the source data in accordance with the requirements of the clinical trial protocols.

To convert data from each clinical assay to international standard units, the WHO international standard for anti-SARS-CoV-2 immunoglobulin (NIBSC code 21/340: https://nibsc.org/documents/ifu/21-340.pdf) and the CEPI standard generated by the UK Medicines and Healthcare products Regulatory Agency (MHRA, former NIBSC) were run in multiple facilities to determine the conversion factor for each assay. The obtained conversion factors for all assays were used to convert the current data (e.g., ELU/mL in ELISA) to International Unit (IU)^11^.

(iv) Participation in external quality assessment programs (EQA) SARS-CoV-2 assay proficiencies across the network are also monitored every 6 months by an external partner, Duke Human Vaccine Institute, through their EQAPOL SARS-CoV-2 Antibody Assay Monitoring (EQAPOL-SAAM) program, and by Q2 Solutions-Nexelis.

Duke University oversees the monitoring of MNA and PNA assay through their SAAM program by providing qualified testing materials consisting of 40 vials of blinded defibrinated plasma samples obtained from National Institute of Allergy and Infectious Diseases (NIAID)-sponsored clinical studies to each of our participating CLN facilities. The 40 samples consist of 10 negative samples and 5 unique samples at 6 replicates that covered a wide range of antibody responses. Testing facilities submit results to the EQA facilitator, who independently analyze the results and generate an assessment report. Testing facilities are scored based on three metrics, namely: (i) Specificity by correctly identifying the ten negative samples, (ii) Equivalence by comparing sample titers to the reference values, and (iii) Precision by comparing the % coefficient of variation (%CV) between sample replicates. All testing facilities within the network receive a performance rating ranging from excellent to fair, signifying the effective development and implementation of clinical neutralization assays.

For the proficiency testing of SARS-CoV-2 ELISA and ELISpot assays, multiple human serum samples and peripheral blood mononuclear cells (PBMCs) were screened after procurement from various biotechnology companies. The selected PBMCs and serum samples with various ranges are distributed as blinded samples to the participating facilities by Q2 Solutions-Nexelis every six months. This distribution schedule ensures regular proficiency testing and allows for ongoing assessment of assay performance across the network. A crucial aspect of the CEPI-CLN mission is to ensure the standardization of assays across our network by using the same key reagents and identical protocols and guidelines across all facilities for each assay. By standardizing assays, we have enhanced our capability to precisely quantify the anti-SARS-CoV-2 immune response. This standardized approach not only facilitates the comparability of results within our network but also enables direct comparisons between various vaccine candidates evaluated in distinct geographic regions.

## Discussion

**Fig. 3:**
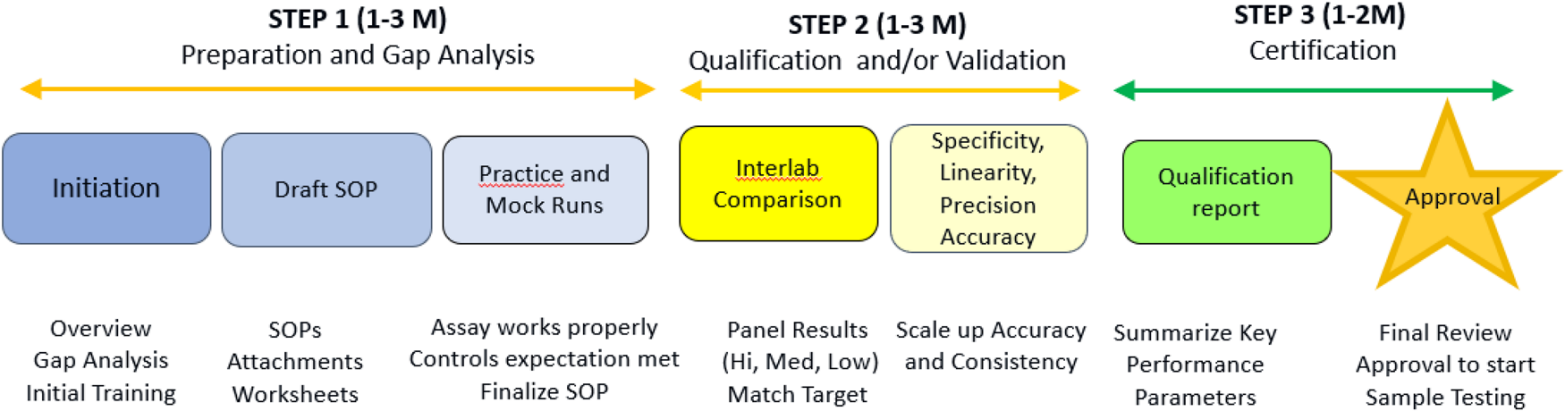
Assay transfer. The development and validation of assays are crucial steps that are performed in the reference facilities to ensure accuracy and reliability. Once the assays are validated, they are transferred to the designated receiving facilities, as illustrated below. The average timeline for the transfer of assays typically ranges between 3 to 8 months. However, it is important to note that this timeline may vary based on several factors, such as obtaining necessary shipping permits, variations in infrastructure or quality systems in facilities, particularly those located in low- and middle-income countries (LMICs), and other considerations.

The successful transfer of standardized assays to the CEPI-CLN has been achieved through the meticulous execution of a well-defined assay transfer plan (Fig. 3). In addition, a post-tech transfer plan has been established to monitor and assess the ongoing performance of the transferred assays within the CEPI-CLN framework.

Despite the significant contribution of the CEPI-CLN to the development of standardized assays for the assessment of COVID candidate vaccines, we have identified areas for improvement. Moving forward, we would like to be better prepared for other potential pandemic or endemic pathogens to rapidly develop appropriate assays that correlate well with protection against the target pathogen, are regulatory compliant, and can be easily deployed within the network to support vaccine development. We have learned from challenges which we encountered during the COVID-19 pandemic, including accessing well-characterized and traceable assay control reagents in sufficient quantities, regional challenges and long delays in the shipment of key critical reagents within the global network, alignment of Quality Management System (QMS) procedures and compliance within the network, establishment of rigorous and continuous monitoring of the network both internally and by an accrediting external body, improving the speed of clinical testing to reduce turn-around times (TAT) and providing data readouts to vaccine developers faster, incorporation of high throughput regulatory compliant technologies within the network, availability of technical expertise, and access to high containment facilities (BSL-3 and BSL-4) for safe handling of biospecimens such as convalescent sera and virus isolates when required to support the development of immunological assays. Efforts are currently underway to address these challenges, leveraging the range of expertise and regional experience across the network to identify and establish suitable solutions. In addition, the combination of a strategic transfer plan and a structured post-tech transfer approach could significantly contribute to the overall success and effectiveness of the assay transfer process. These approaches will be utilized for the immunological evaluation of vaccine candidates against other pathogens in the future.

## Methods

The methodologies for the assays are available in detail in our published article in Lancet Microbes (please refer to reference #9). This article comprehensively describes the assay procedures, including the setup, execution, and analysis methods. For a thorough understanding of the assay methodologies, we recommend referring to this publication.

## Data Availability

Requests for access to the control data can be submitted to the CEPI-CLN Project Manager, Mr. Vijay Zala, at vjay.zala@cepi.net. Please note that the clinical trial results have not been discussed in this article and cannot be shared due to confidentiality and privacy protection regulations. The SARS-CoV-2 assay protocols can only be shared within the CEPI-CLN network based on the signed frame agreement during the SARS-CoV-2 pandemic. However, assay protocols for other diseases in the future will be made available to everyone.
